# ‘Trying to pin down jelly’ - exploring intuitive processes in quality assessment for meta-ethnography

**DOI:** 10.1186/1471-2288-13-46

**Published:** 2013-03-21

**Authors:** Francine Toye, Kate Seers, Nick Allcock, Michelle Briggs, Eloise Carr, JoyAnn Andrews, Karen Barker

**Affiliations:** 1 Nuffield Orthopaedic Centre, Oxford University Hospitals NHS Trust, Oxford, UK; 2 Royal College of Nursing Research institute, School of Health & Social Studies, University of Warwick, Coventry, UK; 3 Faculty of Medicine & Health Sciences School of Nursing, Midwifery and Physiotherapy, University of Nottingham, Nottinghamshire, UK; 4 School of Health and Life Sciences, Glasgow Caledonian University, Cowcaddens Road, Glasgow, UK; 5 Institute of Health and Well Being, Leeds Metropolitan University, Leeds, UK; 6 Faculty of Nursing, University of Calgary, Alberta, Canada

**Keywords:** Quality appraisal, Qualitative, Qualitative synthesis, Meta-ethnography

## Abstract

**Background:**

Studies that systematically search for and synthesise qualitative research are becoming more evident in health care, and they can make an important contribution to patient care. However, there is still no agreement as to whether, or how we should appraise studies for inclusion. We aimed to explore the intuitive processes that determined the ‘quality’ of qualitative research for inclusion in qualitative research syntheses. We were particularly interested to explore the way that knowledge was constructed.

**Methods:**

We used qualitative methods to explore the process of quality appraisal within a team of seven qualitative researchers funded to undertake a meta-ethnography of chronic non-malignant musculoskeletal pain. Team discussions took place monthly between October 2010 and June 2012 and were recorded and transcribed. Data was coded and organised using constant comparative method. The development of our conceptual analysis was both iterative and collaborative. The strength of this team approach to quality came from open and honest discussion, where team members felt free to agree, disagree, or change their position within the safety of the group.

**Results:**

We suggest two core facets of quality for inclusion in meta-ethnography - (1) *Conceptual clarity;* how clearly has the author articulated a concept that facilitates theoretical insight. (2) *Interpretive rigour;* fundamentally, can the interpretation ‘be trusted?’ Our findings showed that three important categories help the reader to judge interpretive rigour: (ii) What is the context of the interpretation? (ii) How inductive is the interpretation? (iii) Has the researcher challenged their interpretation?

**Conclusions:**

We highlight that methods alone do not determine the quality of research for inclusion into a meta-ethnography. The strength of a concept and its capacity to facilitate theoretical insight is integral to meta-ethnography, and arguably to the quality of research. However, we suggest that to be judged ‘good enough’ there also needs to be some assurance that qualitative findings are more than simply anecdotal. Although our conceptual model was developed specifically for meta-ethnography, it may be transferable to other research methodologies.

## Background

Assessing whether the research we are reading is good is often a challenge [1]. Some would argue that quality appraisal is never compatible with qualitative research methodology. However, if we want to use qualitative research to inform clinical practice, we need to be confident that the research *is* good enough. Although there is much debate about how, or whether, to assess the quality of qualitative research [2,3] there is little doubt that we *do* make judgements about the quality of research. However, how exactly we make this judgement is not always clear. Although there are now many suggested criteria for appraising the quality of qualitative research, there is no consensus about what makes a qualitative study ‘good’ or ‘good enough’ [2,3]. Suggested criteria for appraising quality vary considerably [4], and appraisal checklists do not seem to produce consistent judgements [3].

The debate about assessing quality is relevant to qualitative research synthesis, which is a growing area of healthcare research. A recent Health Technology Assessment report identified 41 qualitative syntheses [2]. Other reviews of qualitative syntheses suggest that this number is much larger and increasing dramatically [5,6]. Hannes and colleagues demonstrate that the number of qualitative syntheses has doubled within four years. These syntheses are useful, as the proliferation of qualitative studies makes it difficult for clinicians and policy makers to use knowledge from qualitative studies to inform practice and policy [7]. Researchers have begun to consider the impact of ‘quality’ on the qualitative research syntheses [6,8,9], and ask questions such as ‘should we exclude inadequately reported studies?’[9]. In health care research, although there are criteria for rating *quantitative* studies to decide whether research should be included in a systematic review [10], excluding studies on the basis of quality is not so straightforward. A common approach in quantitative research synthesis is to use sensitivity analysis to allow the reviewer to assess the impact of including ‘lower quality’ studies on the conclusions of a synthesis. However, it is a challenge to determine the impact of including studies of diverse quality if we do not agree about what good quality is. Some researchers suggest that we attempt to distinguish ‘fatal flaws’ in qualitative systematic review [3,11]. However, this does not bring us closer to deciphering or articulating what constitutes a ‘fatal flaw’. Importantly, if we aim to stand up to critics and improve the quality of our research, we need to continually challenge our interpretation of quality, and place it under scrutiny [12]. In short, if we are to use the findings of qualitative research to inform clinical practice the process by which we judge quality should be transparent.

One of the difficulties in appraising quality is that it is partly intuitive, and involves subjective judgement by the reader based on *tacit* knowledge. Polanyi proposes a *tacit* way of knowing the world, whereby we ‘know more than we can tell’ [13]. For Polanyi, tacit knowledge is a form of knowledge that can have value alongside knowledge gained from scientific enquiry. Although ‘tacit’ knowledge is intuitive, researchers have a certain obligation to challenge their intuitive ‘certainty’. This paper explores the intuitive process of quality appraisal within a team of qualitative researchers conducting a meta-ethnography of patients’ experience of chronic non-malignant musculoskeletal pain. We used qualitative methods to explore factors that contributed to our decision that a study was ‘good enough’ to be included in a qualitative synthesis. This process was played out during a project funded by the National Institute of Health Research (NIHR) between 2010 and 2012. Although interpreting quality often felt like *‘trying to pin down jelly’*, we aimed to explore, challenge and articulate our own judgements about what constituted quality in qualitative research. We aim to bring transparency and honesty to the process of determining ‘quality’ and to reach some conclusions about what we decided was ‘good enough’ to be included. To our knowledge, the issue of quality has not been explored using qualitative research methods. We aim to explore what constitutes quality for us as researchers.

### Study context

The research team comprised seven qualitative researchers with a track record of publication in qualitative and quantitative research, including research synthesis. The team was funded by the NIHR Health Services and Delivery Research (HS&DR) programme to produce a meta-ethnography of patients’ perceptions of chronic non-malignant musculoskeletal pain. Meta-ethnography is one method used to synthesise the findings from qualitative research [14]. It is currently the most widely cited method of qualitative synthesis in health care research [2,15], and has been used in several areas of health research [2,5,16-18]. Meta-ethnography is an *interpretive* rather than aggregate form of knowledge synthesis. In other words, it aims to develop conceptual understanding, rather than provide an aggregate account of findings. Analysis involves translating the concepts of qualitative research, and exploring how these concepts are related to each other; thus ‘translating qualitative studies into one another’ [14]. By comparing translations, meta-ethnography aims to provide a deeper conceptual understanding of a particular phenomenon. Studies must therefore provide sufficient description of conceptual categories to allow translation [14]. The team needed to decide what studies were ‘good enough’ to include in the synthesis. We used three methods of appraisal to provide a starting point for discussion. Firstly, we used the questions developed by the Critical Appraisal Skills Programme (CASP) [19] to appraise qualitative research, which have been used for appraising the quality of studies for inclusion in meta-ethnography [16,18,20]. Secondly, as two team members were experienced in using the Qualitative Assessment and Review Instrument (JBI-QARI) designed by the Joanna Briggs Institute to manage, appraise, extract and synthesise qualitative data as part of a systematic review of evidence [21], we used this alongside the CASP to stimulate discussion. Finally, we categorised papers as either: a ‘key paper’ (KP) that is ‘conceptually rich and could potentially make an important contribution to the synthesis’; a satisfactory paper (SAT); fatally flawed (FF); irrelevant to the synthesis [3]. This method has also been used to determine inclusion of studies into meta-ethnography [17]. The concepts FF, SAT and KP have not been defined, but are global judgements made by a particular appraiser that are likely to comprise several (unspecified) factors. Irrelevant studies are those that do not explore the topic chosen for the review. Between November 2010 and March 2012, we appraised 93 relevant qualitative studies for inclusion in the meta-ethnography. Early in the process of appraisal we found that the categories ‘KP’ and ‘FF’ were not mutually exclusive i.e. some studies that we judged to be’ fatally flawed’ were simultaneously judged as ‘KP’ because they were conceptually rich or insightful. It therefore became clear that we needed to ‘unpick’ what we meant by FF, SAT and KP.

CASP – 10 questions for appraising qualitative research [19]

1. Was there a clear statement of the aims of the research?

2. Is a qualitative methodology appropriate?

3. Was the research design appropriate to address the aims of the research?

4. Was the recruitment strategy appropriate to the aims of the research?

5. Were the data collected in a way that addressed the research issue?

6. Has the relationship between researcher and participants been adequately considered?

7. Have ethical issues been taken into consideration?

8. Was the data analysis sufficiently rigorous?

9. Is there a clear statement of findings?

10. How valuable is the research?

JBI- QARI questions [21]

1. Is there congruity between the stated philosophical perspective and the research methodology?

2. Is there congruity between the research methodology and the research question or objectives?

3. Is there congruity between the research methodology and the methods used to collect data?

4. Is there congruity between the research methodology and the representation and analysis of data?

5. Is there congruity between the research methodology and the interpretation of results?

6. Is there a statement locating the researcher culturally or theoretically?

7. Is the influence of the researcher on the research, and vice versa, addressed?

8. Are participants, and their voices, adequately represented?

9. Is the research ethical according to current criteria or, for recent studies, and is there evidence of ethical approval by an appropriate body?

10. Do the conclusions drawn in the research report flow from the analysis, or interpretation, of the data?

## Methods

Team meetings took place monthly between October 2010 and June 2012 and were recorded and transcribed. Each team member wrote a ‘positionality statement’ at the beginning and end of the study. This encouraged us to reflect on our position regarding qualitative rigour and quality. FT kept research notes of all meetings. All notes, transcripts and statements were uploaded onto Nvivo 9, a computerised software package developed to assist analysis of qualitative data. FT coded the data to reflect the team members’ interpretation of quality. FT then grouped these codes into categories that made sense of the data. She presented these categories regularly at team meetings, where they were discussed and modified to arrive at the final conceptual analysis of quality. The developing interpretation was presented back to the team during on-going meetings and all members had the chance to make comments throughout. Quality became a topic that we discussed throughout the two year duration of the project and discussions remained open and lively. The development of our conceptual analysis was both iterative and collaborative. The strength of the team approach to quality came from open and honest discussion, where team members felt free to agree, disagree, or change their position within the safety of the group. We illustrate our conceptualisation of quality using excerpts from team discussions.

## Results and discussion - an interpretation of quality

At the outset, the team had concerns that using checklists for appraising the quality of studies would not necessarily improve the quality of the meta-ethnography.

The more precise you try to be about it . . . you lose the intuitiveness out of that process and it starts to become more of a criteria related . . . you end up measuring what you can measure rather than what is necessarily important . . . you destroy the flower by dissecting it

**Figure 1:**
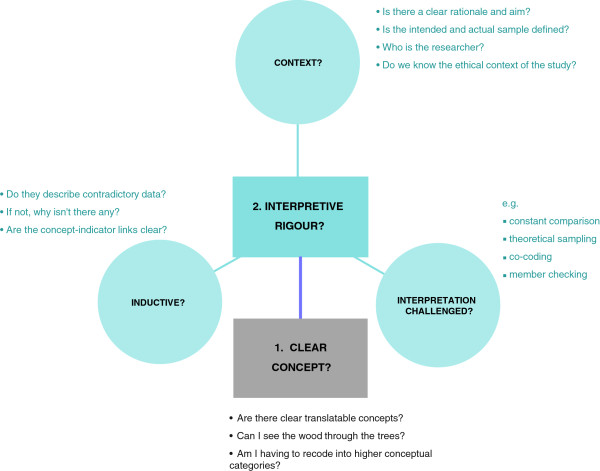
Summary of team’s conceptual approach to quality appraisal for meta-ethnography.

However, through discussion over two years, we developed an interpretation of ‘quality’ within the team. This interpretation is specific to our appraisal of quality for a meta-ethnography but could be transferable to other areas involving quality appraisal. Figure 1 summarises our conceptual approach to quality appraisal for meta-ethnography. We suggest two core facets of quality for inclusion in meta-ethnography (1) *Conceptual clarity* (how clearly has the author articulated a concept that facilitates theoretical insight), and (2) *Interpretive rigour* (what is the context of interpretation; how inductive are the findings; has the interpretation been challenged?) Although we have chosen certain quotes to illustrate our interpretation, all team members contributed to the conceptualisation of quality. Team members remain anonymous in quotations.

This process highlights the dynamic nature of knowledge construction. For example, at the outset one team member did not agree that clear methodological reporting was a facet of quality for qualitative research, or that appraising quality was possible, or useful.

There are so many influential anthropological pieces on pain and chronic illness; Byron Good . . . Arthur Kleinman . . . that do not report *any* method but are clearly rich and influence my thoughts and actions.

I am in the process of my own dialectic here; on the one side I can’t see how you can do it (appraise quality), and on the other I know that it has to be done somehow. I realise that ‘*anything does not go*’.

Until now, I have been on the side that say it is virtually impossible to appraise qualitative research, (although at the same time felt it my own inadequacy for only doing this ‘by gut’). Being in this team has made me realise that I am drawn in by the readability and seductiveness of a research piece.

The following interpretation illustrates how this view transformed over the two years. This is only one possible interpretation of quality, although one that we found useful.

1. Conceptual clarity

The first key facet of quality integral to meta-ethnography is the presence and clarity of concepts for translation. This conceptual clarity goes beyond the author’s report of research method. We could argue that this is integral to qualitative research beyond evidence synthesis. However, conceptual clarity is integral to meta-ethnography which requires clear concepts as data. The team agreed that inclusion would ultimately be determined by whether or not we could decipher a translatable concept. The challenge of this is deciding what a concept is, as it can be difficult to decipher a concept through the description; in other words, to see ‘the wood through the trees’.

It is really difficult to describe *depth* in terms of qualitative research . . . you read something and you have a real sense that there is a depth of analysis, but if someone asks me describe the difference between something quite superficial and something with real depth . . . I am struggling . . . its intuitive, I know what I mean, but I am not able to articulate it.

Comments from appraisal notes included: ‘findings generic; no clear concepts; will be difficult to translate without further analysis’; ‘analysis not finished; very descriptive account’; ‘seemed more descriptive than analytical’; ‘no conceptual analysis’; ‘basic content analysis with no theoretical insight’; ‘highly descriptive’; ‘there were just big splodges of quotes without any sort of interpretation’.

I am finding quite a lot that are kind of generic titles and no coherent constructs reported . . . for meta-ethnography you have to have some sort of clear construct to be able to take away . . . I am reading this thinking, are we actually going to be able to do anything with this?

Sometimes they are categories rather than themes; just a group of things together.

We agreed that one of the aims of qualitative analysis is to develop categories that help us to understand an experience, rather than just describe that experience. In this way, conceptual categories do more than just *describe* the data; they contribute to the development of ideas. Qualitative analysis aims to suggest categories that order and explain the data, thereby ‘constructing a theoretical language grounded in instances of data’ [22]:[89]. In some studies, these categories appear more descriptive, whereas in others, they appear more conceptual. For the purposes of qualitative research, we could describe a concept as a meaningful *idea* that develops by comparing particular instances and make sense of them through categorisation. In this way, Holton describes grounded theory coding as the ‘act of conceptual abstraction’ that ‘isolates a part or aspect of an entity or phenomenon for the purposes of contemplation’ [23]. Fundamentally, a concept must *explain* not just *describe* the data. However, the act of description itself requires a level of interpretation, and it can be difficult to distinguish between a descriptive and a conceptual account. It may not be useful to see description and conceptualisation as dichotomous, but as two poles on a continuum.

I think it is hard [for students] to know the difference between something that is a conceptual category and something that is descriptive category.

You can’t just describe . . . you are conceptualising when you are writing a paragraph about twenty patients that you have interviewed . . . even with descriptive phenomenology . . . you still are interpreting aren’t you? . . . You have to . . . otherwise you can’t communicate an idea, unless we conceptualise things, can we?

In the end the reader must make a personal judgment about whether they think there is a translatable concept or not; one reader might see a concept whereas another might see no more than description. There are no clear cut rules. A practical application of this concept would be: if you find yourself thinking about recoding and condensing the findings of a qualitative study into higher conceptual categories in order to help you make sense of them, then the conceptual analysis is lacking. In these circumstances, we concluded that there was no useable concept for the meta-ethnography, even if the methods were thought to be well reported.

This raises another important issue; although we regarded conceptual clarity as an important facet of quality for meta-ethnography, is this enough to determine the quality of primary studies to be included in a meta-ethnography? In other words, should we include studies purely on the basis of their conceptual strength?

I think we are quite certain that method was absent really . . . but several of us have put it down as fatally flawed and still a key paper ’cos it is very conceptually rich.

Quality is more than sound methods, and a clear report of method does not make something conceptually insightful. Through discussion, we agreed that for the purposes of meta-ethnography studies must report methods well enough.

I can see what you are saying that it is conceptually rich but on the other hand there is so little to draw on methodologically to know whether any of the conceptual richness is grounded in any data.

To be honest I feel a bit uncomfortable that actually those concepts have come from something that we think is methodologically flawed . . . I almost feel you have to make a decision; if you feel that concepts they are coming out with are not supported adequately . . . I think you have to exclude them.

Although we found that in some cases we felt intuitively that some studies were likely to have been methodologically ‘good enough’, we could only make a judgement based on the published account. Even if some studies were more engaging and readable than others, a well written captivating piece of research might not allow the reader to judge the adequacy of the methods.

You could be seduced by really good writing based on a crappy study. It has got to be good enough.

There is a lethal cocktail of poor methodological study with a lack of interpretation that is incredibly well written . . . that is a lethal cocktail, cos it can be incredibly emotive and instil lots of passion.

2. Interpretive rigour - a reflexive research approach to research

Interpretive rigour encapsulates a reflexive approach to research; we all agreed that *reflexivity* was an integral facet of quality. A reflexive study demonstrates ‘a self-questioning’ approach [22], and the reader should get a sense that reflexivity pervades all aspects of the study. Whereas modern scientific research rests on the assumption that repeatable and unbiased method will result in *true* findings [24], our interpretation is based on the premise that knowledge is a constructed interpretation of the world. We agreed that high quality research must demonstrate that the researcher has been interpretively rigorous [25]:[205]. The reader needs to judge the quality of the authors’ interpretation, rather than method per se. Fundamentally, can the interpretation ‘be trusted?’ Our findings showed three important categories that demonstrate a reflexive approach: (a) What is the context of the interpretation? (b) How inductive is the interpretation? (c) Has the researcher challenged their interpretation?

(a) What is the context of interpretation?

To judge quality, the reader needs to know the context in which the interpretation was made. This information is situational and will usually be located within the methods section of a paper. There are several aspects of method that we felt helped the reader to judge the context of the researcher’s interpretation: a clear rationale and aim; definition of the intended and actual sample; a reflexive statement locating the researcher; a description of the ethical relationship between researcher and participants.

There will always be a relationship between what we are aiming to find and what we actually find. A clear statement of rationale and aim therefore contributes to a reflexive account of the research process by describing the context of the interpretation. In short, we cannot assess interpretive rigour without knowing the aim at the outset of the study. The relative importance of providing a clear aim differed within the team and shifted over the year.

I suppose I didn’t think at first [that having a clear aim] was a quality issue, because you might not actually find things that were related to the aim that you originally had . . . you might go in looking for something and come out finding something completely else.

It is not necessarily the aim itself that is important . . . it was about how the aim might shape and drive the approach to the research, and therefore the whole findings that would derive from it.

Your aim . . . is almost part of your reflexivity isn’t it, cos if you are going out to look for something it is going to affect what you find.

From the outset, the team also agreed that a clear definition of the sample was an important contextual factor. This allows the reader to judge whether or not the sample served the research purpose, and how transferable the findings are to other contexts. The reader needs to judge: Is the context of the sample adequately described? Does the sample match the intended aim? Does the sample influence the findings? Comments from team appraisal notes included: ‘[I was] not left with idea of who the sample are and how they were recruited’; ‘[there were] no details on why some groups were rejected’; ‘sample not ideal for self-management strategies as not self-managing’; ‘not clear where women were from or what type of treatment they were having’.

There is no data at all about what the other forty participants were like and any issues around context setting . . . we have got no way of knowing how they made their decisions about how these particular six were recruited, they have just said, ‘believe us that these six were exemplars’.

The team agreed that a clear description of both the intended and the *actual* sample was necessary.

You might define what your sample is going to be . . . but the really important thing is that you know more about people that end up in the study . . . it’s about making the decision of how do I apply this to the context I am working in?

We also agreed that *who* the researcher is will inevitably effect their interpretation, and the reader needs to be able to judge the significance of this: with the proviso that a statement of *who* the researcher is *per se* does not give the account authority [22] or necessarily help the reader to make a sound judgement.

If you said, ‘I am from a nursing background’, what does that tell you? . . . from a reflexivity point of view is pretty limited. The reaction you get if someone says, ‘what do you do?’ and you say your background is nursing . . . you can almost predict what their reaction is going to be . . . I think there is an assumption about a whole set of things that goes on.

If I say I am a physiotherapist and if say I am an anthropologist I have quite different reactions from people . . . it is more about picking out what the such-and-such views are, rather than what *I am*.

A reflexive account gives the reader an insight into the researcher’s perspective and therefore provides important context. Knowledge is inherently constructed from a ‘viewpoint’ or ‘bias’ and reflexivity provides a statement of this. It should be available (as far as possible) to the reader, so that they can make judgements about how this viewpoint affects the researcher’s interpretation. It can also provide an insight into the ethical relationship between researcher and participant. Virtually none of the qualitative studies that we reviewed incorporated a reflexive statement, even though this features on appraisal checklists. A researcher’s perspective may come from personal or professional factors, for example, a psychologist may (or may not) draw different conclusions to an anthropologist. Comments from appraisal notes included: ‘low on reflexivity although authors sympathies apparent throughout’; ‘focuses primarily on psychology’; ‘recognise reflexivity but no account of personal reflexivity and impact on findings’. Although the team agreed that a reflexive statement was an important facet of interpretive rigour, we only agreed that one study extensively addressed the issue of reflexivity (CASP question 6 - Has the relationship between researcher and participants been adequately considered?). Team members also described experiences of being asked to remove reflexive statements by journal reviewers or editors.

A description of the ethical relationship between researcher and participants also provides the context to allow the reader to judge interpretive rigour. Although we agreed that clearly, research must meet ethical standards, the concept of ethics as a facet of quality was challenged and developed over time, thus demonstrating the dialectic process within the team.

If they haven’t got ethical approval, should we not actually look at it because it is not an ethical study?

I have always taken that stand that ethics wouldn’t be a *quality* issue . . . and this [paper] has actually changed my mind . . . because if there is not an ethical relationship between the research and researched then you are not going to get good data.

In our final interpretation, we agreed that ethical relationship between researcher and participant is an important facet of ‘good study’; for example, a researcher is less likely to get meaningful or ‘true’ data if the relationship is imbalanced, or if they have a particular agenda? This relationship incorporates conflict of interest, power dynamics, impact of researcher on ‘data’ and manipulative reporting. Very few of the studies that we reviewed discussed this relationship. This is particularly interesting in view of the finding that it appears as a criterion on qualitative appraisal tools. A high quality report should therefore give insight into the ethical relationship between the researcher and participants. For example, is there any obvious power discrepancy that will have an impact on findings? Does the relationship between the researcher and the participant affect the findings? (e.g. are they their clinician?) Does the researcher have a vested interest in a particular finding? This ethical relationship was linked to the concept of reflexivity. It is through reflexivity that we may come to challenge our ethical position within the research process

Manipulative reporting or really good reporting to get a point across, isn’t an ethical relationship with the data . . . there are two things that are appallingly badly reported in qualitative research; ethics is one in terms of ethical decisions, and reflexivity and those two things are linked.

A report produced on behalf of the Cabinet office [26] for appraising the quality of qualitative research, supports this emphasis on the relationship between the researcher and participants as a key component of quality, and the importance of an ethical relationship with participants. They also emphasise the importance of reflexivity and transparency as a mark of quality. A practical application of this concept would be: am I getting a sense of where this interpretation is coming from? If so, does this adversely affect the findings?

(b) How inductive is the interpretation?

The team agreed that for the purposes of meta-ethnography another important facet of interpretive rigour is whether or not we feel that the interpretation is grounded in the data (inductive), or is imposed on the data (deductive). Paradoxically, we recognised that, as categorisation of data is inevitably influenced by a priori concepts, a purely inductive stance is logically untenable.

Things aren’t [purely] inductive, there is always a continuum.

I think it is a spectrum . . . it is the whole grounded theory argument, can we bracket off? . . . of course we can’t, but we still have to . . . I don’t think there is a lot of point doing a bit of research unless we are going to be drawing something from what we have observed or heard.

This tension between inductive and deductive approaches goes beyond decisions on appraising quality for research synthesis [27]. A resolution is unlikely whilst we continue to consider *induction* and *deduction* as dichotomous. The reader has to decide whether or not the research is grounded in the data, whilst acknowledging that researchers inevitably utilise prior knowledge in order to make sense of new information. Although it seems unlikely that findings *emerge* from data inductively, we agreed that good research should not impose a priori structures on data; ‘good’ qualitative research should be ‘more inductive’. However, we acknowledged that it can be difficult to decipher whether something is inductive or not. Some approaches (for example studies that developed a framework for analysis from literature review) are explicitly more deductive, but this is not always the case. For the purposes of this qualitative synthesis, we agreed that studies reporting deductive methods did not meet the standard for inclusion. Of particular importance to our interpretation of quality is that the reader needs to be able to judge whether data has been *cherry picked* to support a priori views.

Two particular aspects might help the reader to judge how inductive a piece of research is: Has the researcher discussed contradictory data (or suggested why there is none)? Is there a clear link between the proposed concept and the data used to illustrate this theme (clear concept-indicator link)? Absence of contradictory data or *negative voices* might raise concerns about the inductiveness of a piece of research. The reader needs to have confidence that the researcher has not just omitted data because it does not fit their interpretation. Comments from appraisal notes included: ‘Missed negative cases completely; want to find benefits of treatment and found them; what about those who had not resolved, readjusted or redefined; ‘missing voices; lacks resonance - patients *do* experience emotional distress; not all learn to ‘cope’; negative cases lacking’ ‘where are negative voices?’ The significance of reporting contradictory data and recognising that there might be a missing voice was highlighted by team discussions about typologies:

A typology might be useful but people are probably much more fluid than that suggests.

There is a tendency to develop a theory about how patients progress as if it is not messy . . . and they all go differently, and go backwards and forwards . . . [laugh] if only life was so simple.

Recognising the missing voice is linked to the concept of resonance; does the research make sense in the light of your own experience? Are there any obvious unheard voices? In other words, do the findings have face-value?

I read one where they were talking about spinal cord injury, and everyone seemed quite happy with what had happened, it just didn’t ring true . . . obviously there were some unheard voices.

However, resonance as a mark of quality in itself has some inherent flaws. Although a non-resonant piece of research might ring alarm bells, high quality research might challenge rather than resonate with our existing views and thus provide new food for thought. We cannot assume that resonant views are the right ones, and therefore did not include resonance as a marker of quality.

Sometimes there might be a really good report that really didn’t resonate with what we knew, but it was really important because it had new stuff that you hadn’t thought about . . . and therefore why doubt something just because it is not your personal experience.

We agreed that in order to judge the inductiveness of research, the reader also needs to see a clear link between the data and the development of conceptual categories. The reader needs to judge whether the chosen excerpts adequately represent the concept. In other words, does the author provide good ‘concept indicators links’ [22]?

The important thing to me is that there are good concept-indicator links; does the little bit that they have put in there actually describe the concept or do I have to start recoding.

As meta-ethnography relies on translating concepts from original research, we agreed that we should resist the temptation to recode any primary data used by the originating author to illustrate their concepts. Qualitative analysis is the researcher’s interpretation of what is meaningful based on their analysis of the entire dataset. The researcher then chooses excerpts because of their power to explain this interpretation [22]. One could therefore argue that recoding excerpts without knowledge of the entire dataset is methodologically flawed.

As I was reading the quotes I found myself recoding them in my head . . . you think, ‘well I wouldn’t have said that’ . . . I think to a certain extent you have got to say, are the methods in there enough that you are confident that they would have got those concepts in a robust way.

I think that is right because . . . the body of evidence that obviously the researcher has looked at and is picking out quotes from, may more adequately support what they are actually saying and that is always really difficult from an extracted version.

If the concept-indicator link is not clear then the reader cannot judge the inductiveness of the interpretation. Quality assessment should allow the reader to trust that the interpretation has been derived in a rigorous way from the data. A practical application of this would concept would be: does there seem to be a missing voice? Does the quotation provided support the finding that the author has described?

(c) Has the researcher challenged their interpretation?

A quality account should also demonstrate how the researcher has challenged their interpretation of the data by looking at it from other points of view. The term ‘triangulation’ has come into disrepute in qualitative circles, and criticised for assuming a positivist stance. It refers to the process of using multiple ways of looking at the same thing in order to *converge* on the truth [28]. However, the aim of considering alternative views is not necessarily to agree on an interpretation, but rather to enter into a dialectic process whereby your ideas are challenged and modified. This can lead to greater conceptual insight by challenging the boundaries of our own interpretations; just as a single word from another person can jog our memory or spark off insight where we had not expected it.

It isn’t checking it is co-creation in a sense of getting into a point of better clarity, distilling almost . . . whereas it is reported as a sense of, well ‘I got someone else to look at my data and therefore what I have chosen is right’.

I have changed here ’cos I have come from a point of view that my interpretation is as good as anyone else’s, but now I think by questioning your own interpretation by getting other views, you are actually moving your interpretation forward, you are not trying to agree.

There are many ways of considering alternative points of view: constant comparison aims to look at the phenomenon from multiple angles [22,26]; theoretical sampling seeks out particular samples in order to challenge developing theories [29]; member checking or respondent validation invites participants to comment on the researcher’s interpretation [30]; co-coding by another researcher aims at collaborative rigour. Although independent coding by another researcher or respondent validation have become popular means of ensuring rigour, this is not always practical, nor integral to good quality research. The idea that collaboration intrinsically improves the quality of research is problematic. For example, what do we do if the collaborator does not agree with our interpretation? Also, people’s perspectives change over time and context, so how do researchers deal with changes in perspective from the time of data collection? In the final analysis, the interpretation, although co-constructed, belongs to the researcher and need not necessarily agree with others.

Member checking does certain things but it doesn’t necessarily give you rigour . . . If you are member checking with someone from whom you have got the data then I think it is a problem cos they might change their minds for a number of different reasons . . . if you ask an individual whether they agree with your generic interpretation they can’t possibly know the whole data on which you have based that.

It is almost the notion that a collaborative rigour or a second person somehow increases the validity but you could argue that what it might do is take you further down a particular perspective . . . so all you are doing is making more certain that you are up a creak.

A practical application of this concept would be: is there any alternative interpretation of the findings possible that we could consider?

## Conclusions

This paper explores the process of determining quality within a team of qualitative researchers conducting a meta-ethnography of chronic non-malignant musculoskeletal pain. This process challenged our ‘intuitive certainty’ and was the focus of knowledge reconstruction. Our aim is to make this process of learning and knowledge construction available to scrutiny from the research community, in the hope of bringing the debate on quality forward.

Two core facets of quality for the purposes of meta-ethnography developed. Firstly, can the reader can see the wood for the trees; is there a clear concept for translation or does the reader find themselves mentally recoding and further abstracting the material presented? Even though descriptive studies require a certain level on conceptual abstraction, there is little doubt that some studies are more conceptual than others. For meta-ethnography, clear concepts are required; however, judgements about whether or not there is a clear concept are made by the reader alone. Secondly, does the study demonstrate interpretive rigour by presenting a reflexive account of the research process? Fundamentally, the reader needs to judge the quality of the authors’ *interpretation;* although sound method is integral to sound interpretation, the quality of any interpretation goes beyond methods per se. We agreed that important facets of interpretive rigour are: how does the context of the research effect the interpretation made? Does the author show how the findings are supported by the data? Does the researcher show how they have challenged their interpretation?

Some argue that excluding studies from qualitative research syntheses on the basis of methodological criteria may mean that insightful studies are excluded [2]. Methodological flaws can be easier to ‘pin down’ and therefore tend to be picked up in quality appraisal. This raises an important issue for quality appraisal that is transferable to quantitative research synthesis - one of the central challenges of explicitly ‘judging’ quality is that it comprises *both* insightfulness and method. Insightfulness and methodological rigour are not necessarily mutually exclusive, but are different facets of quality. Some authors argue that there may be a positive relationship between sound methodological reporting and positive contribution to the synthesis [9], whilst others suggest studies that there may be a negative relationship [2]. We therefore need to consider the relative contribution of methodological and conceptual quality to the process of quality appraisal for qualitative synthesis, and beyond.

Another issue raised is whether we should appraise qualitative studies to determine whether or not to include them in a meta-ethnography? Campbell, et al. (2011) suggest that ‘inclusion of poorer studies is unlikely to have a very distorting impact on qualitative synthesis’ [2]:[45], and suggest that the time spent on quality appraisal might perhaps be better spent. They chose to include conceptually rich studies with a poor report of method, arguing that meta-ethnography is concerned primarily with conceptual development. In contrast to this, Dixon-Woods and colleagues suggest that we attempt to distinguish ‘fatal flaws’ in qualitative systematic review [3,11]. Carroll and colleagues used sensitivity analysis to show the possible benefits of quality appraisal for qualitative research synthesis [9]. Although we excluded studies from our meta-ethnography on the basis that the methodological report was insufficient to make a judgement on interpretive rigour, this does not necessarily mean that these studies were poor, but that there was insufficient information to make a judgment about the interpretation presented. The process of appraisal was extremely time-consuming, and we also need to ask whether or not this is time well spent. It is also important to consider how we answer criticism from other researchers if we include studies with flawed (or not described) methods in our reviews, even if they are conceptually rich.

It is not surprising that qualitative researchers do not agree about whether or not to exclude studies on the basis of quality, as, over and above any epistemological arguments about *multiple realities*, there is no agreement about what quality is. We chose to use several checklists as these checklists are commonly used and were a useful focus for our discussion on quality. These checklists are not the focus of this paper, and we do not necessarily recommend their use, nor would we necessarily use them in subsequent syntheses. It would be interesting to compare our interpretation with criteria in existing checklists, and this could be the focus of further study. We remain convinced that current checklists produce inconsistent judgments. However, analysis of potential reasons for variation is lacking, and this would be an extremely interesting area of research. Qualitative researchers cannot ignore the debate about quality, and their intuitive certainty should not remain sacrosanct; in other words, it is not good enough to say that we ‘know’ quality when we see it. For this reason, we aimed to explore our own intuitive knowledge. We do not expect our views to remain unchallenged, and indeed hope that it will contribute to on-going lively debate.

Importantly our research raises an issue that is transferable to quantitative research; quality comprises *both* method and conceptual insight. In the long run, the epistemological debates of qualitative research are not necessarily pertinent to clinicians making daily decisions about whether to incorporate qualitative findings into their practice. Clinicians and policy makers need practical advice on using qualitative research findings and need to know if it is ‘good enough’. The issue of how to determine quality in qualitative research has continuing relevance. The concept of quality is constructed and dynamic. The criteria by which we judge quality are not fixed but shift and change over time and in relation to context [31]. To a certain extent we need to ‘learn to live with uncertainty and contingency’ [12]:[884], but at the same time challenge and modify our interpretation in the light of collaborative experience. Using qualitative research methods, we aimed to explore and understand the intuitive processes of quality appraisal within a team of qualitative researchers. We present a conceptual model of quality that focuses on (1) conceptual clarity and (2) interpretive rigour. We highlight that methods alone do not determine the quality of research for inclusion into a meta-ethnography. Concepts that facilitate theoretical insight are the raw data of meta-ethnography, and arguably, are integral to the quality of research. However, to be judged ‘good enough’ we suggest that there needs to be some assurance that the interpretation presented is more than simply anecdotal. Although our conceptual model was developed specifically for meta-ethnography, it may be transferable to other research methodologies.

## Competing interests

This project was funded by the NIHR Health Services and Delivery Research programme (project number 09/2001/09).

The views and opinions expressed therein are those of the authors and do not necessarily reflect those of the NIHR HS&DR programme, NIHR, NHS or the Department of Health.

## Authors’ contributions

All authors contributed to the development of the conceptual model and read and approved the manuscript. FT transcribed and coded the data and drafted this manuscript. KS helped to draft the manuscript.

## Authors’ information

FT has a master’s degree in Archaeology and Anthropology and is also a qualified physiotherapist with an interest in chronic pain management. She has expertise and interest in qualitative health research and methodology. KS has a quantitative and qualitative health and pain research background and expertise, and has used mixed methods in most of her research. Her professional background is nursing. NA is a doctoral qualified nurse academic and practising pain nurse. MB has broad experience in systematic reviews. She has completed syntheses of qualitative research using Joanna Briggs QARI methodology. Her professional background is nursing. EC qualified as nurse and throughout her twenty five year research career has utilised mixed methods in her pain research. JA has a background in the social sciences, and post-doctoral experience of conducting qualitative research. KB is a qualified physiotherapist, with experience of running chronic pain management programmes.

## Pre-publication history

The pre-publication history for this paper can be accessed here:

http://www.biomedcentral.com/1471-2288/13/46/prepub
